# A novel leptin receptor antagonist uncouples leptin’s metabolic and immune functions

**DOI:** 10.1007/s00018-019-03004-9

**Published:** 2019-01-18

**Authors:** Lennart Zabeau, Joris Wauman, Julie Dam, Sandra Van Lint, Elianne Burg, Jennifer De Geest, Elke Rogge, Anisia Silva, Ralf Jockers, Jan Tavernier

**Affiliations:** 1 Faculty of Medicine and Health Sciences, VIB-UGent Center for Medical Biotechnology, Flanders Institute for Biotechnology, Ghent University, A. Baertsoenkaai 3, 9000 Ghent, Belgium; 2 Inserm U1016, CNRS UMR 8104, Univ. Paris Descartes, Sorbonne Paris Cité, Institut Cochin, 22 rue Méchain, 75014 Paris, France

**Keywords:** Leptin, Leptin receptor, Receptor cross talk, Uncoupling, Metabolism, Immunity

## Abstract

Leptin links body energy stores to high energy demanding processes like reproduction and immunity. Based on leptin’s role in autoimmune diseases and cancer, several leptin and leptin receptor (LR) antagonists have been developed, but these intrinsically lead to unwanted weight gain. Here, we report on the uncoupling of leptin’s metabolic and immune functions based on the cross talk with the epidermal growth factor receptor (EGFR). We show that both receptors spontaneously interact and, remarkably, that this complex can partially overrule the lack of LR activation by a leptin antagonistic mutein. Moreover, this leptin mutant induces EGFR phosphorylation comparable to wild-type leptin. Exploiting this non-canonical leptin signalling pathway, we identified a camelid single-domain antibody that selectively inhibits this LR-EGFR cross talk without interfering with homotypic LR signalling. Administration in vivo showed that this single-domain antibody did not interfere with leptin’s metabolic functions, but could reverse the leptin-driven protection against starvation-induced thymic and splenic atrophy. These findings offer new opportunities for the design and clinical application of selective leptin and LR antagonists that avoid unwanted metabolic side effects.

**Electronic supplementary material:**

The online version of this article (10.1007/s00018-019-03004-9) contains supplementary material, which is available to authorized users.

## Introduction

The crucial role of leptin in long-term body weight control is well established. This cytokine with hormone-like characteristics is mainly, but not exclusively, produced and secreted by adipocytes, and plasma leptin levels positively correlate with body fat energy stores [1, 2]. Loss-of-function mutations in the leptin-encoding *ob* gene give rise to a complex syndrome that includes not only morbid obesity, but also abnormalities in lipid and glucose metabolism [3], haematopoiesis [4], innate and adaptive immunity (leading to increased risk of infection in mice and men) [5–9], reproduction [10], development [11], angiogenesis [12], vascular remodelling [13], blood pressure [14], and bone formation [15]. Indeed, leptin is more than just a satiety signal and rather acts as a ‘metabolic switch’ that connects the body’s energy stores to high energy demanding processes like immunity and reproduction [16, 17]. In innate immunity, leptin promotes secretion of inflammatory cytokines and the activation of macrophages, neutrophils and natural killer cells [18]. Functions in adaptive immunity include thymic homeostasis, naïve CD4^+^ cell proliferation, promotion of T helper 1 (T_H_1) responses and suppression of CD4^+^CD25^high^ regulatory T cells (Tregs) [18]. Consequently, leptin can contribute to the onset and progression of several T cell-controlled autoimmune diseases, including Crohn’s disease [19, 20], rheumatoid arthritis [21, 22], multiple sclerosis [23, 24] and autoimmune hepatitis [25–27]. Furthermore, clinical reports unequivocally link elevated serum leptin levels (caused by obesity) to an increased risk of certain cancers including prostate [28], breast [29, 30], colorectal [31], renal cancers [32] and multiple myeloma [33, 34].

Leptin mediates its effects upon binding and activation of the single-membrane spanning leptin receptor (LR), encoded by the *db* gene [35], and belonging to the class I cytokine receptor family. Its ectodomain is composed of two cytokine receptor homology (CRH1 and CRH2) domains, which are separated by an immunoglobulin-like domain (IGD) and is followed by two membrane-proximal fibronectin type III (FN III) domains. The CRH2 domain is necessary and sufficient for leptin binding [36, 37], but functional receptor clustering requires interaction with the IGD since leptin site III mutants that fail to contact this domain behave as leptin antagonists [38, 39]. Like all class I cytokine receptors, the LR lacks intrinsic kinase activity and relies for signalling on constitutively associated Janus tyrosine kinases (JAKs) [40]. LR clustering results in JAK trans-phosphorylation and activation of several intracellular signalling cascades including the STAT (signal transducer and activator of transcription), MAPK (mitogen-activated protein kinase), PI3K (phosphatidylinositide 3-kinases) and mTOR pathways [41, 42].

In a recent study, we demonstrated the possible uncoupling of leptin’s metabolic and immune functions at the LR level [43]. Indeed, the interaction between leptin’s site III (consisting of residues S120 and T121) and the LR IGD domain is strictly necessary for the control of body weight and metabolism, but is dispensable for immune signalling. We hypothesised that this non-canonical LR signalling may depend on receptor cross talk mechanisms, as documented for the insulin-like growth factor-I receptor (IGF-IR) [44, 45], epidermal growth factor receptor (EGFR) [46, 47] or the estrogen receptor-alpha (ERα) [48, 49].

Here, we identified a cross talk between LR and EGFR, defined the structural requirements for this cross talk and used this information to design a LR antagonist that selectively interferes with the immunomodulatory effects of leptin without interfering with the canonical LR signalling that controls the metabolic function.

## Materials and methods

### Cell culture, transfection and reagents

Hek293T (human embryonic kidney) cells (ATCC) were grown in DMEM (Dulbecco’s modified Eagle’s medium; Gibco, life technologies) with 4500 mg/l glucose and 10% FCS (foetal calf serum; Invitrogen) in a 10% CO_2_ humidified atmosphere at 37 °C. Cells were transfected with standard calcium phosphate precipitation. Recombinant mouse leptin and leptin S120A-T121A were purified and refolded from *E. coli* inclusion bodies [50]. To prolong the half-life in circulation, leptin was pegylated with a 40 kDa PEG (polyethylene glycol) variant (Sunbright) according to the manufacturer’s instructions. Generation and screening of a mouse leptin receptor-specific VHH library has been described previously [50]. Mono- and bispecific (genetically fused to the sequence of anti-mouse serum albumin (mAlb) via a flexible GGS-linker) VHHs were cloned in the pHEN6C vector for periplasmic *E. coli* expression. Proteins were purified after osmotic shock using the C-terminal 6*His-tag with the TALON metal affinity resin (Clontech). Imidazole (Merck) was used for elution and removed using PD-10 gel filtration columns (GE Healthcare). Protein concentrations were determined using the absorbance at 280 nm (Nanodrop; ThermoFisher Scientific) and purity via SDS-PAGE. Endotoxins were washed away during purification with 0.5% EMPIGEN (Calbiochem, Millipore) and 0.5% CHAPS (Sigma-Aldrich) and afterwards quantified using limulus amoebocyte lysate (LAL) QCL-1000 (Lonza). If still present, endotoxins were removed using Pierce Endotoxin Removal Resin (Thermo Scientific).

### Co-immunoprecipitation

Hek293T cells were transfected with tagged EGFR, LR, LR-FATT variants or combinations thereof. 24 h later, cells were lysed in modified RIPA buffer and cleared lysates were subjected to immunoprecipitation with anti-FLAG M2 affinity resin (Sigma). Proteins in cell lysates (input) and after co-immunoprecipitation were analysed using SDS-PAGE and Western blotting using mouse anti-FLAG (Sigma) and mouse anti-E-tag antibodies (Bethyl), diluted according to the manufacturer’s guidelines in StartingBlock blocking buffer (Pierce) supplemented with 0.1% Tween-20, and revealed using the SuperSignal West Pico Chemiluminescent Substrate (Pierce) after incubation with HRP-conjugated anti-mouse secondary antibody (Jackson ImmunoResearch).

### Luciferase reporter assay

Hek293T cells were transiently transfected with (a combination of) receptors and the STAT3-responsive pXP2d2-rPAP1 (rat pancreatitis associated protein 1)-luciferase reporter [51]. Transfected cells were stimulated overnight as indicated. Cell lysates were prepared [lysis buffer: 25 mM Tris/HCL, pH 7.8, 2 mM EDTA, 2 mM DTT (dithiothreitol), 10% glycerol and 1% Triton X-100], and 35 µl of luciferase substrate buffer [20 mM Tricine, 1.07 mM (MgCO_3_)_4_ Mg(OH)_2_·5H_2_O, 2.67 mM MgSO_4_·7H_2_O, 0.1 mM EDTA, 33.3 mM DTT, 270 µM coenzyme A, 470 µM luciferin and 530 µM ATP, final pH 7.8] was added per 50 µl of lysate. Light emission was measured for 5 s in a TopCount chemiluminescence counter (Packard).

### EGFR phosphorylation

EGFR phosphorylation was analysed in transfected Hek293T cells that were serum starved overnight and left untreated or stimulated with indicated amounts of leptin or leptin antagonist for 5 min. Lysates were prepared in Laemmli loading buffer and proteins were blotted overnight. Phosphorylated and total protein levels were visualised using rabbit anti-phospho-EGFR (Tyr1068) or EGFR antibodies (both Cell Signalling Technology) and an HRP-conjugated anti-rabbit secondary antibody (Jackson ImmunoResearch) as described earlier.

### Time-resolved fluorescence energy transfer (TR-FRET) measurement

The TR-FRET saturation curve is generated in conditions where the expression of the energy donor (EGFR) is kept constant with increasing expression of the energy acceptor (LR) [52, 53]. Hek293 cells were therefore transfected with plasmid vectors encoding SNAP-tagged EGFR (10 ng), and HALO-tagged LR (0–170 ng) constructs (completed to 200 ng/well of 96-well plate with noncoding plasmid) were plated in polyornithine-coated 96-well white plates. Two days after transfection, adherent cells were washed and incubated with Taglite buffer (Cisbio Bioassay) containing 200 nM HALO-Tb and/or SNAP-d2 or SNAP-Tb substrates (Cisbio Bioassay) for 1 h at 4 °C. Cells were washed four times with Taglite buffer and the fluorescence of Lumi4-Tb (excitation at 340 nm, emission at 620 nm, 150 μs delay, and 500 μs integration time) and the TR-FRET signals (340 nm, 665 nm, 150 μs, and 500 μs) were read using an Infinite F500 spectrofluorimeter (Tecan). Background signals were measured on cells transfected with empty vectors and subtracted to obtain the specific signal.

### Kinase substrate sensor (KISS) experiments

The extracellular and transmembrane domains of the EGFR, LR, LR-FATT and the granulocyte-colony stimulating factor receptor (G-CSFR) were fused as prey proteins to the cytoplasmic tail of glycoprotein 130 (gp130), or as bait proteins to a C-terminal fragment of human Tyk2 (Aa 589–1187) as described in [54]. In a standard KISS experiment, combinations of equal amounts of bait and prey DNAs together with the STAT3 responsive pXP2d2-rPAP1-luciferase reporter were transiently transfected in Hek293T cells [54]. Luciferase activity was measured 2 days after transfection as described earlier.

### Plate-binding assays

#### Domain mapping

Specificity of the different VHHs was determined in a plate-binding assay using a series of mouse LR extracellular subdomains (or a combination thereof) fused to secreted alkaline phosphatase (SEAP) [50]. VHHs were bound via a penta-His antibody (Qiagen) on Maxisorp plates (Costar). Excess unbound VHH was washed away with washing buffer (PBS + 0.05% Tween-20) and plates were blocked (PBS + 0.1% Casein) and further incubated with conditioned medium containing LR-SEAP fusions for 2 h at room temperature. After 4 successive washes, bound SEAP activity was measured using the chemiluminescent CSPD substrate (PhosphaLight, Tropix) in a TopCount chemiluminescence counter (Canberra Packard).

#### Affinity measurements

Affinities of the different VHHs for the LR were determined in a similar plate-binding assay as described before: VHH-coated plates were incubated with a serial dilution of the mouse LR extracellular domain fused to SEAP (mLR_EC_-SEAP) [50] for 2 h. The GraphPad Prism software was used to calculate the affinities from the bound SEAP measurements.

#### Serum VHH quantification

Penta-His-coated plates were incubated with a serial dilution of purified VHH as a standard, or a 1:1000 dilution of mouse serum samples for 2 h, and subsequently allowed to bind mLR_EC_-SEAP. Serum VHH concentrations were calculated from the standard curves using the GraphPad Prism software.

### In vivo experiments

Mice were maintained in pathogen-free conditions in a temperature-controlled environment with 12/12 h light/dark cycles and received food and water ad libitum.

To evaluate the metabolic effects, 8-week-old female C57BL/6 mice were treated with 200 µg/mouse/day of mAlb fusions of the selected VHHs intraperitoneally (i.p.) or with PBS for 2 weeks. On a daily basis, animals were weighed and food intake (average food intake per cage/number of mice in cage) and body temperature were monitored. At the end of the experiment, blood was collected for the measurement of VHH concentrations (see above) and serum leptin levels using the mouse leptin DuoSet ELISA (R&D Systems Europe Ltd). Further haematological analysis was performed using a Hemavet 950FS (Drew Scientific) whole blood counter. From each group, one mouse was used for a glucose tolerance test: mice were fasted for 6 h, i.p. injected with 2 g glucose/kg body weight and glucose levels were measured from tail vein blood at the indicated time points using AlphaTRAK 2 Blood Glucose Test Strips (Abbott). After animals were killed via CO_2_ asphyxiation, subcutaneous and visceral fat tissue was dissected and weighed.

To evaluate immune-related effects, 8-week-old male C57BL/6 mice were fed ad libitum or starved for 48 h. The latter group either received a daily i.p. injection of PBS, pegylated leptin (50 µg/mouse/day) or a combination of pegylated leptin (50 µg/mouse/day) and 4.80-mAlb (200 µg/mouse/day). At the end of the experiment, animals were killed via CO_2_ asphyxiation and spleen and thymi were isolated. Organs were weighed and the number of white blood cell, lymphocytes, neutrophils and monocytes in homogenates were quantified using the Hemavet 950FS (Drew Scientific) according to the manufacturer’s guidelines.

### Statistical analysis

Data were analysed with the GraphPad PRISM software using one-way ANOVA followed by Tukey multiple-comparison tests. Results with *P* value less than 0.05 were considered statistically significant.

## Results

### Ligand-independent LR-EGFR clustering

**Fig. 1:**
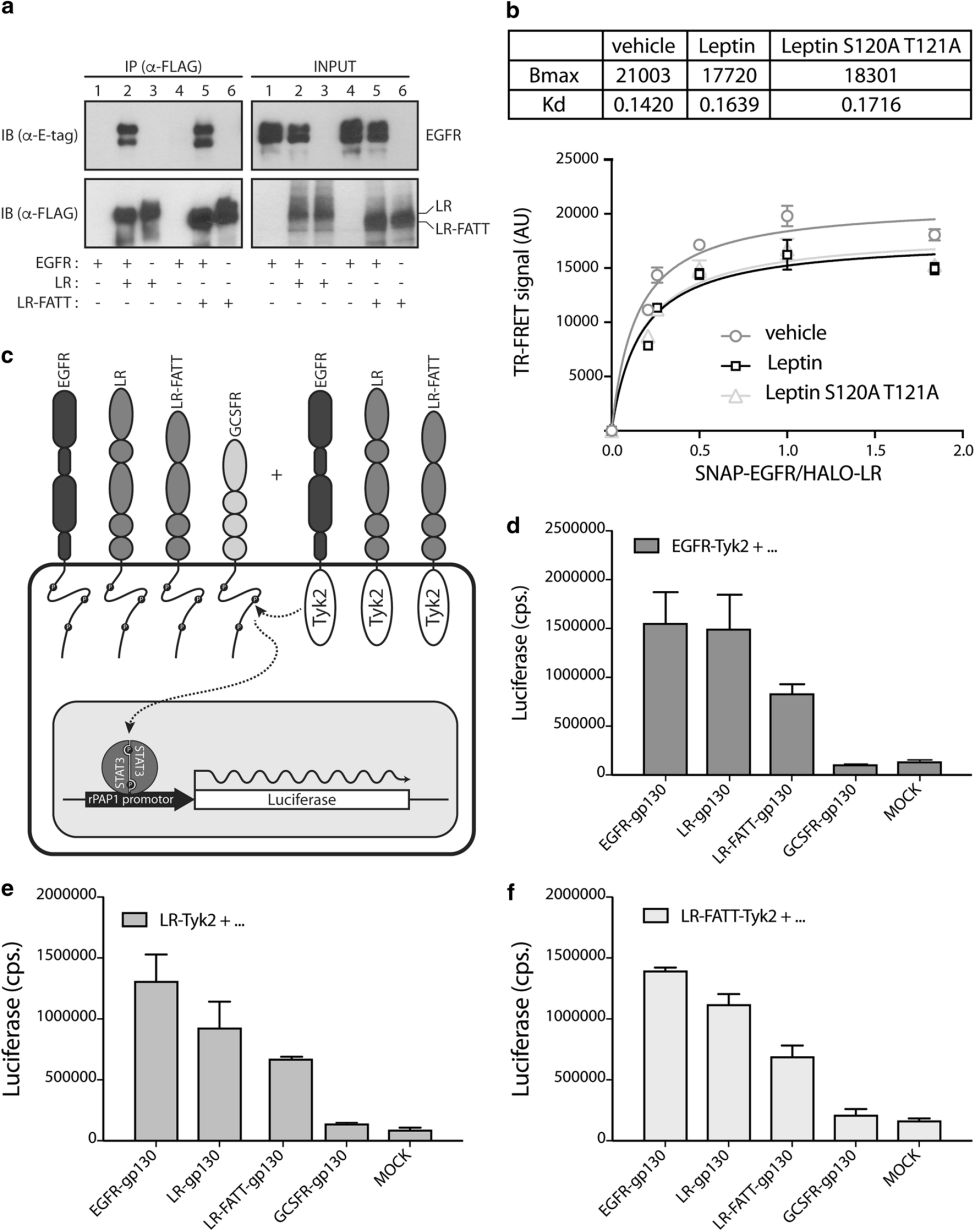
Ligand-independent EGFR-LR clustering does not require the LR IGD domain. **a** EGFR, EGFR + LR/LR-FATT, or LR/LR-FATT transfected cells were lysed and subjected to immunoprecipitation with anti-FLAG beads. Proteins in lysates or after immunoprecipitation were analysed with anti-E-tag and anti-FLAG antibodies in Western blot. **b** TR-FRET saturation experiment assessing the hetero-oligomerization between LR and EGFR at the cell surface of adherent Hek293 cells. TR-FRET intensity at the cell surface, measured in Hek293 cells expressing SNAP-EGFR (labelled with the non-permeable membrane SNAP-Tb substrate) with increasing concentration of HALO-LR (labelled with the non-permeable membrane HALO-d2 substrate), shows a saturation curve reflecting close proximity between HALO-LR and SNAP-EGFR at the cell surface. Stimulation with both leptin and leptin S120A/T121A modifies the TR-FRET signal between LR and EGFR without modification of the TR-FRET_50_ value, suggesting a ligand-induced change of conformation within the hetero-oligomer. **c** Schematic presentation of the KISS setup. **d**–**f** Hek293T cells were transfected with **d** EGFR-Tyk2, **e** LR-Tyk2, or **f** LR-FATT-Tyk2 in combination with gp130 fusion proteins and the STAT3-responsive rPAP1 luciferase reporter. 24 h after transfection, cells were resuspended, seeded in a 96-well plate and cultivated for 2 more days. Data points are the mean (± STDEV) of quadruple luciferase measurements (cps: counts/s). Both datasets are representative for at least three independent experiments

We hypothesised that non-canonical LR signalling (i.e. independent of a correct leptin site III–IGD interaction) might be achieved via cross talk with other receptors like the EGFR. Therefore, we evaluated the role of the IGD in ligand-independent clustering of the LR and EGFR using co-immunoprecipitation, TR-FRET [52, 53] and the mammalian two-hybrid KISS method [54]. In the first setup, we compared the ability of LR-FLAG or LR-FATT-FLAG to precipitate E-tagged EGFR when co-expressed in Hek293T cells. LR-FATT originates from a spontaneous splice mutation causing deletion of the complete LR IGD in all LR isoforms [43]. The observation that both LR and LR-FATT are able to pull down the EGFR (Fig. 1a) indicates that the interaction between both receptors is independent of the presence of LR IGD. To further support the LR-EGFR interaction, we performed additional TR-FRET and KISS experiments in living cells. TR-FRET intensity at the cell surface of Hek293 cells expressing SNAP-tagged EGFR (labelled with the non-permeable membrane SNAP-d2 substrate) with increasing concentration of HALO-tagged LR (labelled with the non-permeable membrane HALO-Tb substrate), shows a saturation curve reflecting close proximity between SNAP-EGFR and HALO-LR at the cell surface (Fig. 1b). In line with the latter, imaging of both receptors expressed in HeLa cells showed co-localisation between cell surface labelled LR and EGFR (Supplementary Figure 1). Both wild-type leptin and leptin S120A/T121A similarly decrease the TR-FRET signal without changing the TR-FRET_50_ value, suggesting that both ligands induce a similar conformational change within the complex (Fig. 1b). In the KISS approach, the extracellular domains of EGFR, LR, LR-FATT or the negative control G-CSFR were fused to the cytoplasmic tails of gp130 or to the kinase and kinase-like domain of Tyk2 (Fig. 1c). The resulting constructs were co-transfected with a STAT3 responsive rPAP1 luciferase reporter which allows detection of the interaction between a gp130- and Tyk2-fused receptor chain upon reconstitution of STAT3 signalling. Luciferase data in Fig. 1d–f clearly illustrate that both EGFR and LR form homo-oligomers on the membrane, but also cluster with each other in the absence of any ligand, and that, in accordance with the co-immunoprecipitation data, this clustering does not depend on the LR IGD.

### Effect of EGFR on non-canonical LR signalling

**Fig. 2:**
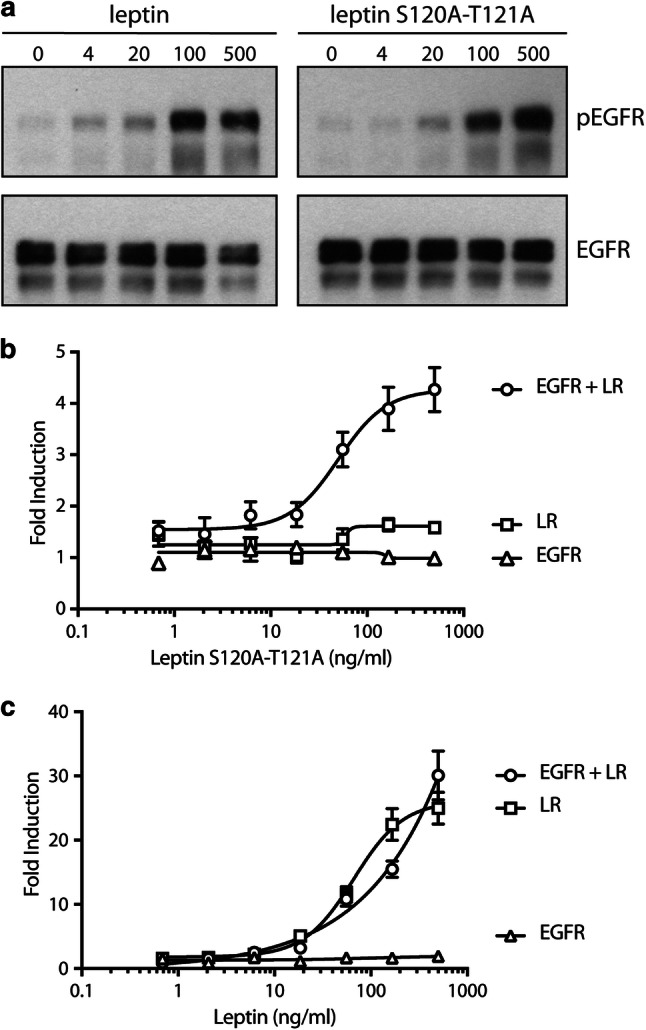
EGFR allows IGD-independent LR signalling. **a** EGFR + LR transfected cells were serum starved overnight, stimulated for 5 min with a serial dilution wild-type or mutant leptin and lysed. Lysates were blotted onto a nitrocellulose membrane and analysed using antibodies specific for phosphorylated (Y1068) EGFR or total EGFR. **b**, **c** EGFR, LR, or the combination, were co-transfected with the rPAP1 luciferase reporter in Hek293T cells. Two days after transfection, cells were stimulated with a serial dilution of leptin S120A-T121A (**b**) or wild-type leptin (**c**) as indicated. Mean luciferase counts (± STDEV.) of quadruple measurements are plotted. Both datasets are representative for at least three independent experiments

The leptin S120A-T121A site III mutant is unable to interact with the LR IGD and therefore unable to initiate canonical JAK2-STAT3 signalling ([38] and Fig. 2b). However, we found that both wild-type leptin and the antagonistic leptin mutein S120A-T121A are able to induce EGFR phosphorylation in a dose-dependent manner in cells expressing both receptors (Fig. 2a). Furthermore, co-expression of the EGFR partially restores signalling of leptin mutein S120A-T121A in a STAT3-responsive luciferase reporter assay (Fig. 2b). In contrast, we could not observe a significant difference in STAT3 signalling mediated by wild-type leptin in LR or LR + EGFR transfected cells (Fig. 2c).

### Interfering with LR-EGFR cross talk

**Fig. 3:**
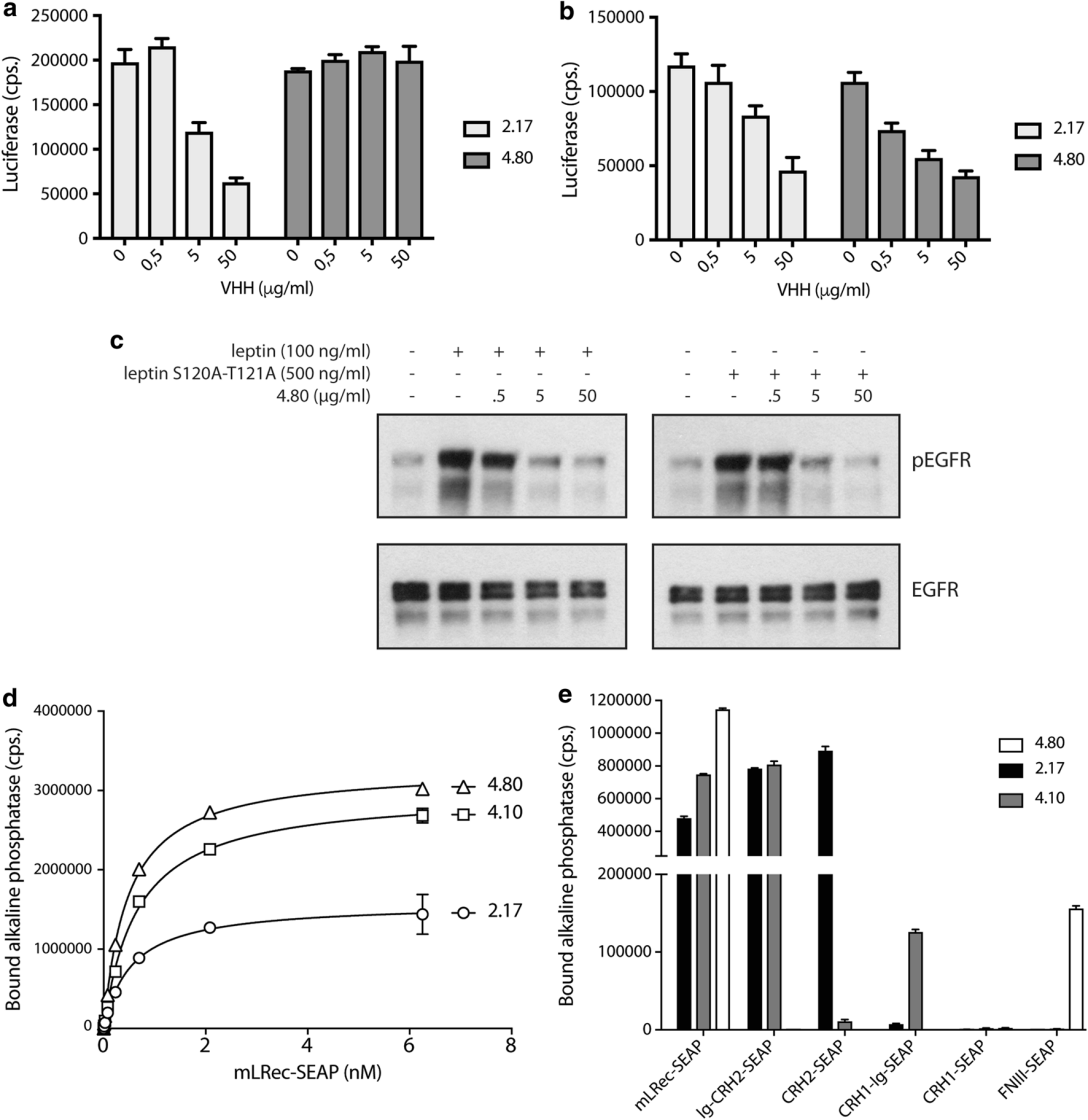
VHH 4.80 selectively interferes with EGFR-LR cross talk, but not with canonical LR signalling. LR (**a**) or EGFR + LR (**b**) expressing cells were stimulated with wild-type leptin or leptin S120A-T121A, respectively, in the presence of a serial dilution 2.17 or 4.80 VHH. Mean luciferase values (± STDEV) of triplicate measurements are plotted. **c** EGFR + LR transfected were stimulated with a sub-optimal concentration of wild-type leptin (100 ng/ml) or leptin S120A-T121A (500 ng/ml) in the presence of increasing concentrations of 4.80 VHH. Phosphorylated and total EGFR levels were visualised using Western blot with specific antibodies. **d** Plates coated with VHH 2.17, 4.10 or 4.80 were incubated with a serial dilution of LR_EC_-SEAP fusion protein. Bound alkaline phosphatase activity (± STDEV) of triplicate measurements is plotted. **e** Specificity of VHHs is determined in a similar manner using SEAP fusion proteins of LR sub-domains

In a previous study, we evaluated a library of LR-specific VHHs for their effect on leptin binding and STAT3-dependent LR signalling [50]. We found that IGD, CRH2, and FN III-specific VHHs can neutralise LR signalling, while only certain CRH2 VHHs can block leptin binding to its receptor. The experimental setup in which the leptin S120A-T121A mutant is able to generate a STAT3-dependent signal in LR-EGFR co-expressing cells (Fig. 2b) prompted us to re-screen this library for binders that could selectively interfere with this LR-EGFR cross talk. VHH 2.17, which blocks the CRH2–leptin interaction [50] interfered, as expected, with both LR and LR-EGFR signalling (Fig. 3a, b). Interestingly, VHH 4.80 specifically interfered with LR-EGFR signalling (Fig. 3b), but not with canonical LR receptor activation (Fig. 3a). In line with the STAT3-dependent reporter data, VHH 4.80 was able to dose-dependently interfere with both wild-type leptin and leptin S120A-T121A-induced EGFR phosphorylation (Fig. 3c).

We applied a plate-binding assay using a mLR_EC_-SEAP fusion protein to determine the affinity of the different VHHs. Figure 3d shows that VHH 4.80 binds the receptor with low nanomolar affinity (0.47 ± 0.017 nM) comparable to VHH 2.17 (0.544 ± 0.065 nM) and VHH 4.10 (a neutralising IGD-specific VHH; 0.66 ± 0.029 nM). A similar plate-binding assay using different LR subdomains or combinations thereof was then used to show that VHH 4.80 binds to the LR FN III domains (Fig. 3e). VHHs 2.17 and 4.10 only bind CRH2 or IGD containing chimeras, respectively.

### Effect of VHH 4.80 on metabolism

**Fig. 4:**
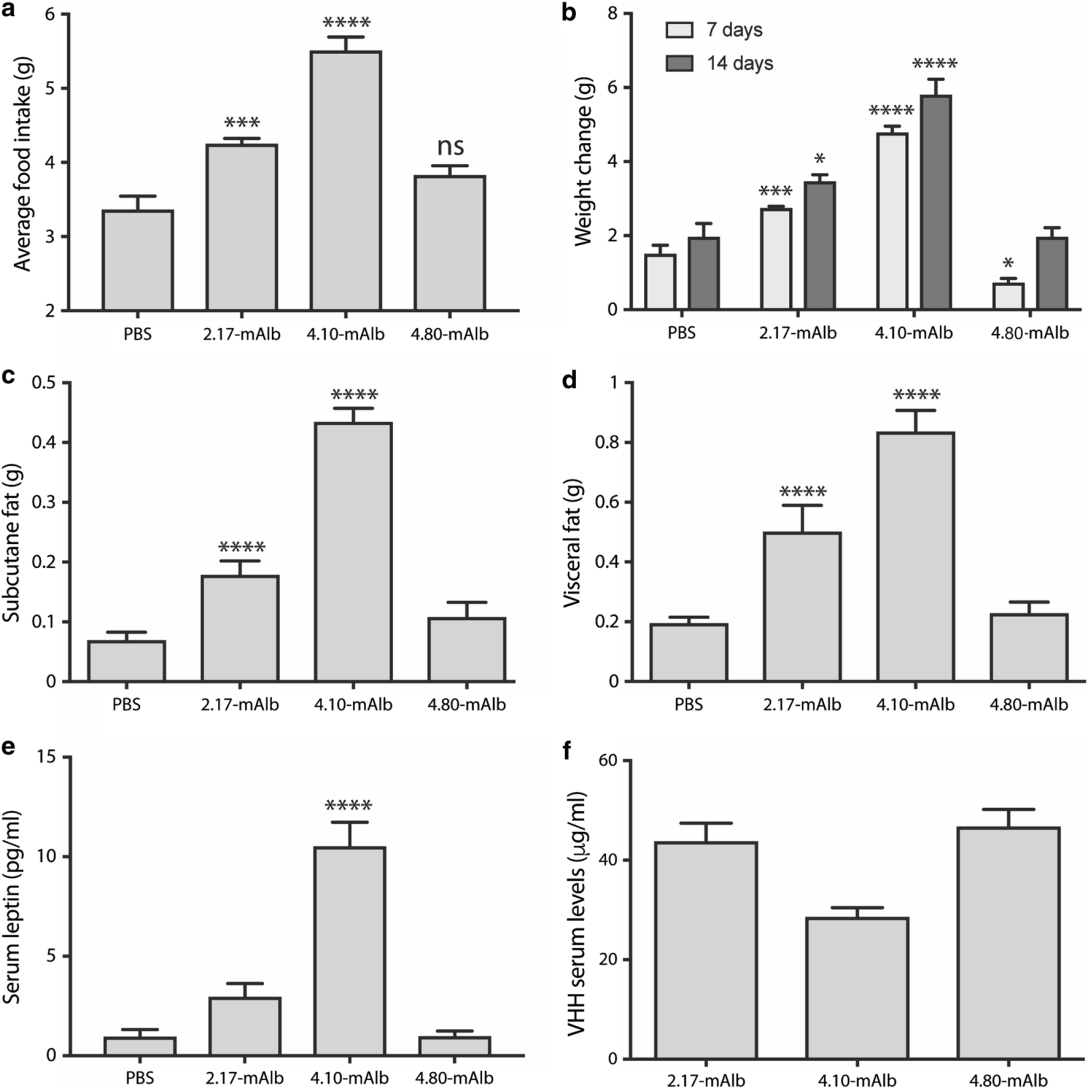
Effect of LR-specific on metabolism. C57BL/6 mice were i.p. injected daily with PBS (*n* = 6) or 200 µg of the mAlb fusions of the LR-specific VHHs 2.17, 4.10 or 4.80 (*n* = 6) for 2 weeks. Daily average food intake (± SEM) was followed (**a**) and mean body weight changes (± SEM) after 7 and 14 days are plotted (**b**). After 2 weeks, animals were killed and subcutaneous (**c**) and visceral fat (**d**) was isolated and weighed (mean ± SEM). Serum leptin (**e**) and VHH (**f**) levels (mean ± SEM) were determined as described in “Materials and methods”. *P < 0.05, **P < 0.01, ***P < 0.005, and ****P < 0.0001 compared to PBS treatment. Data are representative for three independent experiments

VHH 2.17 (CRH2 specific) and 4.10 (IGD specific) both interfere with canonical LR signalling and thus leptin’s metabolic functions as evidenced by the in vivo increase in body weight and other metabolic parameters like fat pads, insulin and glucose levels [50]. In a next experiment, we compared the effects of VHH 4.80 on metabolic parameters with the other two VHHs . To prolong half-life in circulation, VHHs were genetically fused to a VHH directed against mouse serum albumin (mAlb) [50]. Healthy mice were treated with the resulting bispecific VHHs on a daily basis and food intake and body weight were measured throughout the duration of the experiment. Data in Fig. 4a and b show that treatment with 2.17-mAlb and 4.10-mAlb resulted in a significant increase in food intake and body weight, while 4.80-mAlb does not seem to have any effect. Mice were killed on day 14 and fat pads (subcutaneous and visceral) were dissected and weighed. 4.80-mAlb treatment did not result in a significant increase in fat mass, and this again was in contrast to the other two VHHs (Fig. 4c, d). Increase in body weight resulted in hyperleptinaemia in the case of 4.10-mAlb, and to a lesser extent 2.17-mAlb, but not in the case of 4.80-mAlb (Fig. 4e). Basal blood glucose levels did not differ significantly between the different treatments at the end of the study and there was no difference in glucose tolerance between all treatment groups (Supplementary Figure 2a, b). To exclude the possibility that the lack of effects of 4.80-mAlb on metabolism could be due to a different accumulation in circulation, we used the mLR_EC_-SEAP binding assay (see above) to quantify the levels of the bispecific VHHs. Data in Fig. 4f show that 4.80-mAlb (46.66 ± 3.50 μg/ml) levels are comparable to those of 2.17-mAlb (43.72 ± 3.73 µg/ml) or 4.10-mAlb (28.53 ± 1.72 µg/ml). No toxic side effects of the treatments were observed in respect to body temperature, red blood cell numbers, haematocrit and haemoglobin concentration (Supplementary Figure 3a–d).

### Effect of VHH 4.80 on leptin immune signalling

**Fig. 5:**
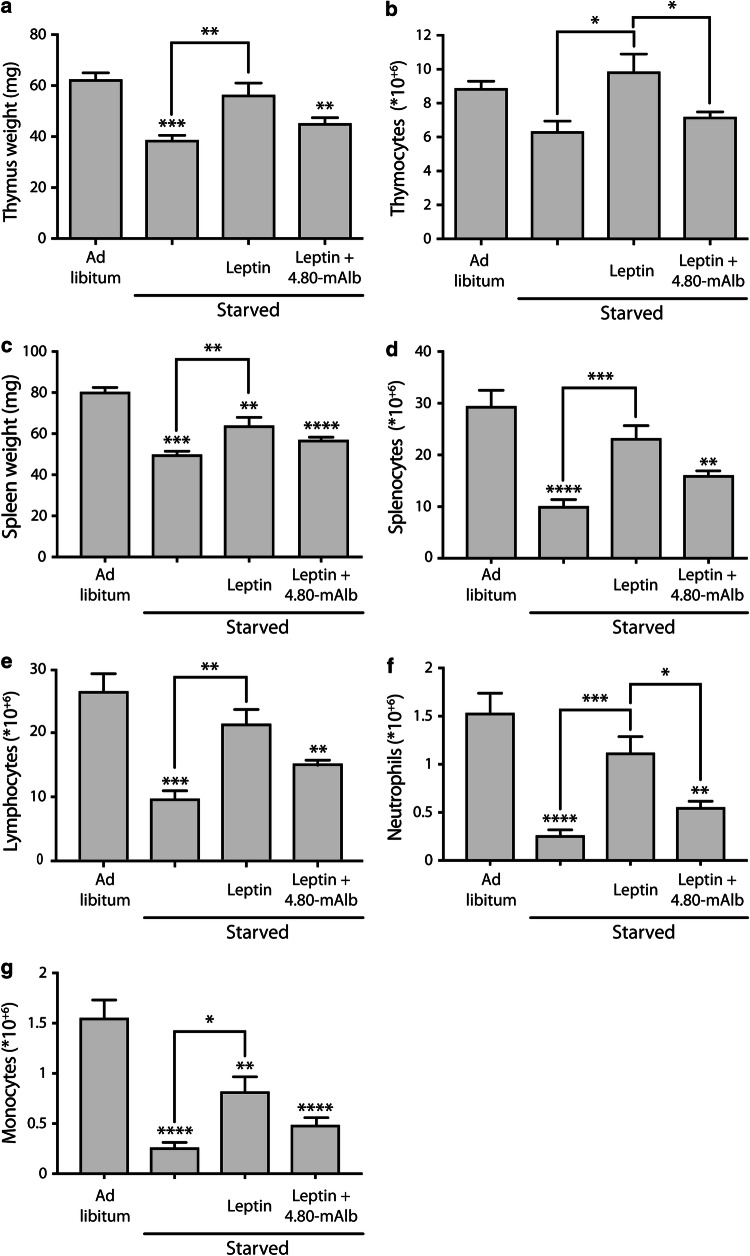
VHH 4.80 blocks leptin protection against starvation-induced splenic and thymic atrophy. C57BL/6 mice were fed ad libitum or were starved for 48 h. Starved animals were injected i.p. at the start of the starvation and 24 h later with PBS, pegylated leptin or a combination of pegylated leptin and 4.80-mAlb. Animals were killed, thymi (**a**, **b**) and spleens (**c**, **d**) isolated and weighed (**a**, **c**) and the total number of white blood cells (**b**, **d**), splenic lymphocytes (**e**), neutrophils (**f**) and monocytes (**g**) counted using the Hemavet instrument. Values represent mean ± SEM; *P < 0.05, **P < 0.01, ***P < 0.005 vs. ad libitum-fed controls, unless stated otherwise. Data representative for three independent experiments

When white blood cell counts in circulation were measured after 2-week treatments with the VHHs (see earlier), we observed a trend towards lymphopaenia (but not neutropaenia) in mice treated with 4.80-mAlb (Supplementary Figure 4). This suggests a possible effect of VHH 4.80 on leptin immune signalling. We therefore examined the effect of the VHH on immune functions in a state of starvation. Starvation is known to negatively affect the weight and cellularity of the immune compartments spleen, thymus and bone marrow [55–57], and this atrophy can be blocked by exogenous administration of leptin [55, 56, 58]. To examine the effect of VHH 4.80 in this experimental setup, 8-week-old mice were starved for 48 h with or without exogenous administration of pegylated leptin. One group also received intraperitoneal injections of 4.80-mAlb. As expected, data in Fig. 5a–d show that starvation resulted in a decreased weight and cellularity of both spleen and thymus. Leptin administration protected against these effects in most cases, while 4.80-mAlb could, at least in part, prevent this protection. Several splenocytic subpopulations were further characterised and a similar interference of 4.80-mAlb on the leptin-induced restoration of lymphocyte, neutrophil and monocyte counts was observed (Fig. 5e–g). These data indicate that 4.80-mAlb interferes with leptin-mediated immune functions. Altogether, our data showed that VHH 4.80 inhibits leptin-mediated LR-EGFR activation and cross talk and that it counteracts leptin-mediated immune responses.

## Discussion

Cytokine receptors often appear as inactive, pre-formed complexes on the cellular surface in the absence of ligands. Well-studied examples include the erythropoietin (Epo) receptor [59], the growth hormone (GH) receptor [60], and the interleukin-6 (IL-6) receptor [61]. Bimolecular fluorescence complementation (BiFC) experiments illustrated not only that all members of the EGFR family form homo- and heterodimers in the absence of their respective ligands, but also that this clustering already occurs in the endoplasmic reticulum (ER) and depends on the intracellular receptor domains [62, 63]. Similarly, the high basal signal in the absence of leptin in bioluminescence resonance energy transfer (BRET) [64] and FRET [65], and co-immunoprecipitation experiments [66, 67] suggest that the LR forms pre-formed dimers (or oligomers) on the cellular surface. This clustering likely involves cysteine residues in the CRH2 domain and, as for the EGFR, already occurs intracellularly [68]. Our co-immunoprecipitation and TR-FRET or adapted KISS experiments in Fig. 1 showed that both the EGFR and LR not only form homodimers (or oligomers), but also heterodimers (or oligomers) with each other on the cellular surface. TR-FRET experiments at the plasma membrane with fluorophores coupled to the N-terminus of the extracellular part of the receptors demonstrate close proximity between the extracellular chains of both receptors (Fig. 1b). Inversely, lack of FRET signal between C-terminal tagged receptors, observed by Eisenberg and colleagues [69], might suggest that the FRET probes on the cytoplasmic domains are likely too distant for the energy transfer. The clustering between LR and EGFR appears to be independent of the LR IGD, since LR-FATT, a naturally occurring and a signalling-deficient LR variant lacking the complete IGD identified in obese *fatt/fatt* mice [43], is as efficient in immunoprecipitating the EGFR as wild-type LR. Likewise, co-expression of EGFR and LR-FATT KISS bait and prey proteins resulted in clear STAT3-dependent signalling.

The EGFR can be activated by its “natural” ligands (including EGF, transforming growth factor-α (TGF-α), heparin-binding EGF-like growth factor, amphiregulin, and epiregulin), but also by cross-activation with G-protein-coupled receptors, the tumour necrosis factor receptor (TNFR), or IGF-IR (reviewed in [70–73]). Activation of the EGFR is widely recognised as a potent signal in the regulation of diverse biological processes including cell proliferation, survival and motility, depending on the cellular system, the stimulus and the repertoire of signalling molecules recruited [74]. Likewise, not only leptin binding, but also cross talk with ERα, IGF-IR, lipoprotein receptor-related protein 1 and 2 (LRP1 and LRP2), and vascular endothelial growth factor (VEGF) receptor has been reported to activate the LR [41].

Cross talk and cross-activation between LR and EGFR is well documented mainly in cancer cell lines, but also in muscle, salivary gland, and mucosal cells and in rat kidneys (reviewed in [41]). Several of these studies showed reciprocal cross-phosphorylation of both receptors and observed that EGFR inhibitors can inhibit leptin-induced signalling events. Both long and short isoforms of the LR were shown to phosphorylate the EGFR family member ErbB2 (both kinase positive and kinase negative) in a leptin-dependent way, suggesting that this cross-activation is independent of downstream LR signalling [69]. The principal function of EGFR is to regulate epithelial tissue development and homeostasis. In pathological settings, such as lung and breast cancer, the EGFR is a driver of tumourigenesis [75]. Aberrant activation of the EGFR and its downstream pro-oncogenic signalling pathways, such as the MAPK and PI3K pathway, promotes cancer cell proliferation, including their chronic initiation and progression through the cell cycle (reviewed by [76]). Leptin was shown to participate in human breast cancer progression and metastasis [77] and induces, via EGFR phosphorylation, clonogenicity, anchorage-independent growth, migration, Notch activation and survivin upregulation in human breast cancer cells [78], illustrating that this LR-EGFR cross talk also has possible biological implications in the context of EGFR signalling.

The interaction between the LR IGD and leptin’s binding site III is essential for the formation of an activated LR complex and downstream signalling. Deletion of this domain results in a receptor completely devoid of biological activity [36, 37]. Likewise, mutation of residues in leptin involved in this interaction resulted in an antagonist both in vitro and in vivo [38, 39]. In strong contrast, we gathered several lines of evidence that LR-mediated immune signalling, but not the control of body weight, can occur in the absence of this interaction: (i) the spontaneous natural S120C mutation in the leptin gene gives rise to high incidence of early-onset obesity in the Nochurli population. Surprisingly, affected patients are still fertile and not more susceptible to infections (unpublished data). This latter is in strong contrast to earlier described human loss-of-function mutations in leptin or LR genes [79]. (ii) *Fatt/fatt* mice carry a spontaneous splice mutation that causes deletion of the complete IGD in all LR isoforms [43]. These animals are hyperphagic and morbidly obese, but, in contrast to other leptin or LR-deficient rodents, display only minimal changes in size and cellularity of the thymus and respond to concavalin A in a model for autoimmune hepatitis. (iii) Treatment of healthy mice with an IGD-specific neutralising VHH induced weight gain and hyperinsulinemia, but failed to block development of experimentally induced autoimmune multiple sclerosis, arthritis and hepatitis [43].

In this study we now show that the leptin S120A-T121A antagonist, which is unable to interact with the LR IGD, is capable of inducing EGFR phosphorylation comparable to wild-type leptin (Fig. 2a). Furthermore, co-expression of EGFR partially restores STAT3-dependent signalling by this mutant through the LR (Fig. 2b). The signalling capacities of the leptin S120A-T121A mutant suggest that the structural requirements for LR signalling vs LR-EGFR cross talk appear to be fundamentally different. To further explore this hypothesis, we evaluated the effect of mouse LR-selective VHHs from a previously designed library [50] on both types of signalling. We found that VHH 4.80, directed against the LR FN III domains, selectively interfered with LR-EGFR cross talk (measured by leptin S120A-T121A-induced STAT3 signalling in LR and EGFR co-transfected cells), but not with canonical LR signalling (leptin stimulation of LR expressing cells)(Fig. 3), while VHH 2.17, which was previously shown to interfere with leptin binding to its cognate receptor [50], clearly blocks both pathways. VHH 4.80 also blocks leptin or leptin S120A-T121A driven EGFR phosphorylation.

In a next step, we compared the effect of the selective VHH 4.80 with previously characterised, neutralising VHHs 2.17 and 4.10 [50] on metabolism in healthy mice. All VHHs were coupled to a mouse serum albumin-specific VHH (mAlb) to prolong half-life in circulation [50]. Both VHH 2.17 and 4.10 treatments resulted in a significant increase in daily food intake, body weight and subcutaneous and visceral fat mass, while a 14-day treatment with VHH 4.80 did not alter these parameters in a significant manner (Fig. 4). This difference could not be explained by a different accumulation of the VHHs in circulation, since VHH 4.80 levels were comparable to VHH 2.17 levels and higher than VHH 4.10 levels after 2 weeks of treatment. No significant differences in basal blood glucose levels and glucose tolerance were observed between the treated groups, which is in line with the study of Levi et al. where 3 days treatment with a pegylated mouse leptin antagonist (L39A/D40A/F41A) did not alter glucose disposal [80].

The LR-EGFR cross talk could provide a molecular explanation for the LR immune signalling in the absence of the IGD interaction. EGFR is known to be critically involved in tissue development and homeostasis as well as in the pathogenesis of cancer. EGFR antagonists were one of the first anti-cancer treatments developed targeting a receptor tyrosine kinase. Several studies illustrate that a substantial part of the clinical responses observed following EGFR targeting treatments may not only be mediated by direct effects on the tumour, but also by regulation of immune responses, an aspect of EGF biology that remains undervalued [81]. For example, the EGFR is expressed on many immune cells including macrophages [82], monocytes [83], plasma cells [84] and some T cell subsets such as effector CD4^+^ T cells and FoxP3-expressing Treg cells [85], thereby guiding the cellular functionality. The observation that VHH 4.80 blocks leptin-driven EGFR phosphorylation and the partial restoration of signalling by leptin S120A-T121A upon LR-EGFR co-expression prompted us to study the role of LR-EGFR cross talk in vivo. We could show that VHH 4.80, at least in part, reverses the leptin-driven protection against starvation-induced thymic and splenic atrophy measured by cellularity and weight of these organs (Fig. 5). In line with the latter, starvation conditions also severely decrease the number of lymphocytes, neutrophils and monocytes, and leptin reverses those effects, while VHH 4.80 interferes with the leptin-induced normalisation of these immune cellular counts.

Together, our data show that the structural requirements for signalling via the LR or LR-EGFR cross talk are fundamentally different. Based on this observation we were able to identify a VHH that selectively inhibits the cross talk, but not the canonical LR activation mechanism. Daily administration of this VHH apparently did not alter any of the tested metabolic parameters, but had significant effects on leptin signalling in the immune compartment as observed in our starvation experiments. Leptin and LR antagonists (leptin mutants, leptin-derived peptides, neutralising antibodies and soluble LR variants) were shown to have a clear potential as therapeutics for the treatment of several autoimmune diseases and cancer [9]. However, the major drawback of most, if not all, current leptin and LR antagonistic strategies is that they also give rise to considerable undesirable weight gain. Uncoupling of leptin’s metabolic and immune functions, for example based on the EGFR cross talk, might revitalise the potential of leptin and LR antagonists as a therapeutic strategy.

### Electronic supplementary material

Below is the link to the electronic supplementary material.

**Supplementary Fig.** **1. Co-localization between LR and EGFR in cells** (**a**) Co-localization of HALO-LR (HALO-d2) and SNAP-EGFR (SNAP-green) labeled at the cell surface of HeLa cells at 4 °C (to prevent constitutive internalization of the receptors). (**b**) Co-localization of HALO-LR (HALO-d2) and SNAP-EGFR (SNAP-green) labeled at the cell surface of HeLa cells and stimulated or not with leptin 20 nM for 30 min at 37 °C. (**c**) Co-localization of SNAP-EGFR (SNAP-green) with leptin-d2 (20 nM) in LR co-expressing HeLa cells, suggesting co-localization between EGFR, LR and Leptin-d2; whereas in the absence of LR co-expression, no leptin-d2 labeling is observed

**Supplementary Fig.** **2. Effect of LR specific VHH’s on basal glucose levels and glucose tolerance** C57BL/6 mice were i.p. injected daily with PBS (n = 6) or 200 μg of the mAlb fusions of the LR specific VHH’s 2.17, 4.10 or 4.80 (n = 6) for two weeks. At the end of the experiment, blood was collected for the measurement of basal blood glucose levels (**a**) and an intraperitoneal glucose tolerance test (IPGTT) (**b**) was performed after six hours of fasting

**Supplementary Fig.** **3. Toxicity assessment of the LR specific VHH’s** No toxic side effects were observed based on body temperature (**a**) during the duration of the experiment or on haematological parameters such as red blood cells numbers (**b**), haematocrit (**c**) and haemoglobin concentration (**d**) at the end of the two-week treatment

**Supplementary Fig.** **4. Analysis of white blood cells (WBC) and WBC subtypes** Two week treatment with VHH 4.80-mAlb (n = 3) causes partial lymphopenia but not neutropenia. Values represent mean ± SEM; *P < .05, **P < .01, ***P < .005

## Abbreviations

BiFC: Bimolecular fluorescence complementation
BRET: Bioluminescence resonance energy transfer
CRH: Cytokine receptor homology
EGFR: Epidermal growth factor receptor
Epo: Erythropoietin
ER: Endoplasmic reticulum
ERalpha: Estrogen receptor-alpha
FN III: Fibronectin type III
FRET: Fluorescence resonance energy transfer
G-CSF: Granulocyte-colony stimulating factor
GH: Growth hormone
gp130: Glycoprotein 130
IGD: Immunoglobulin-like domain
IGF-IR: Insulin-like growth factor-I receptor
IL-6: Interleukin-6
KISS: Kinase substrate sensor
LR: Leptin receptor
LRP: Lipoprotein receptor-related protein
MAPK: Mitogen-activated kinase
PEG: Polyethylene glycol
PI3K: Phosphatidylinositide 3-kinase
rPAP: Rat pancreatitis associated protein
SEAP: Secreted alkaline phosphatase
STAT: Signal transducer and activator of transcription
TGF-alpha: Transforming growth factor-alpha
TNF: Tumour necrosis factor
TR-FRET: Time-resolved fluorescence energy transfer
VEGF: Vascular endothelial growth factor
VHH: Variable domain of heavy chain antibody
