# Supplementary figures and images for: A novel leptin receptor antagonist uncouples leptin’s metabolic and immune functions
Source: Cell Mol Life Sci. 2019 Jan 18;76(6):1201–14. doi: 10.1007/s00018-019-03004-9 (PMC11105424; doi:10.1007/s00018-019-03004-9)

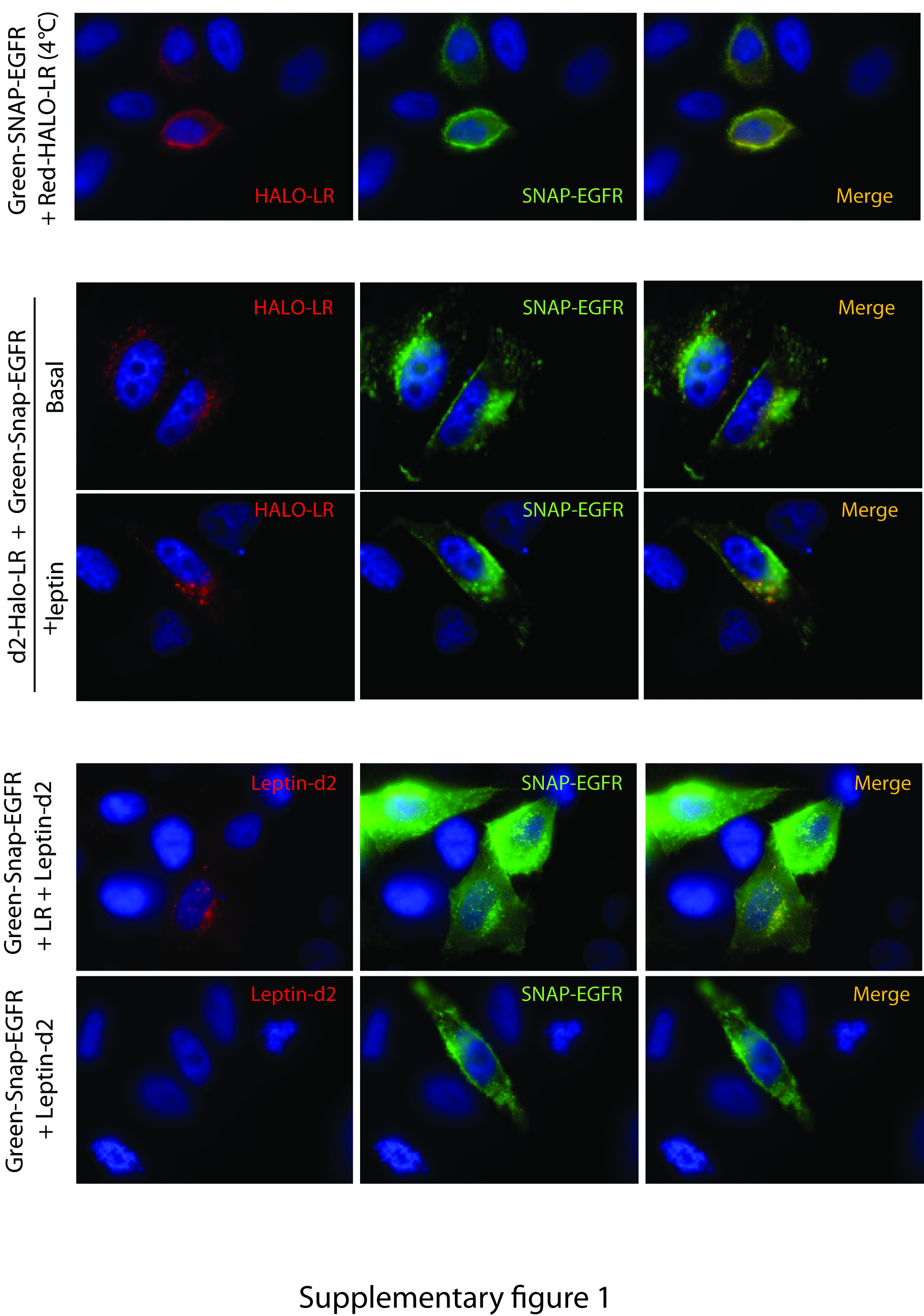

Supplement: Supplementary file 1 — Supplementary Fig. 1. Co-localization between LR and EGFR in cells (a) Co-localization of HALO-LR (HALO-d2) and SNAP-EGFR (SNAP-green) labeled at the cell surface of HeLa cells at 4 °C (to prevent constitutive internalization of the receptors). (b) Co-localization of HALO-LR (HALO-d2) and SNAP-EGFR (SNAP-green) labeled at the cell surface of HeLa cells and stimulated or not with leptin 20 nM for 30 min at 37 °C. (c) Co-localization of SNAP-EGFR (SNAP-green) with leptin-d2 (20 nM) in LR co-expressing HeLa cells, suggesting co-localization between EGFR, LR and Leptin-d2; whereas in the absence of LR co-expression, no leptin-d2 labeling is observed [file 18_2019_3004_MOESM1_ESM.tif]

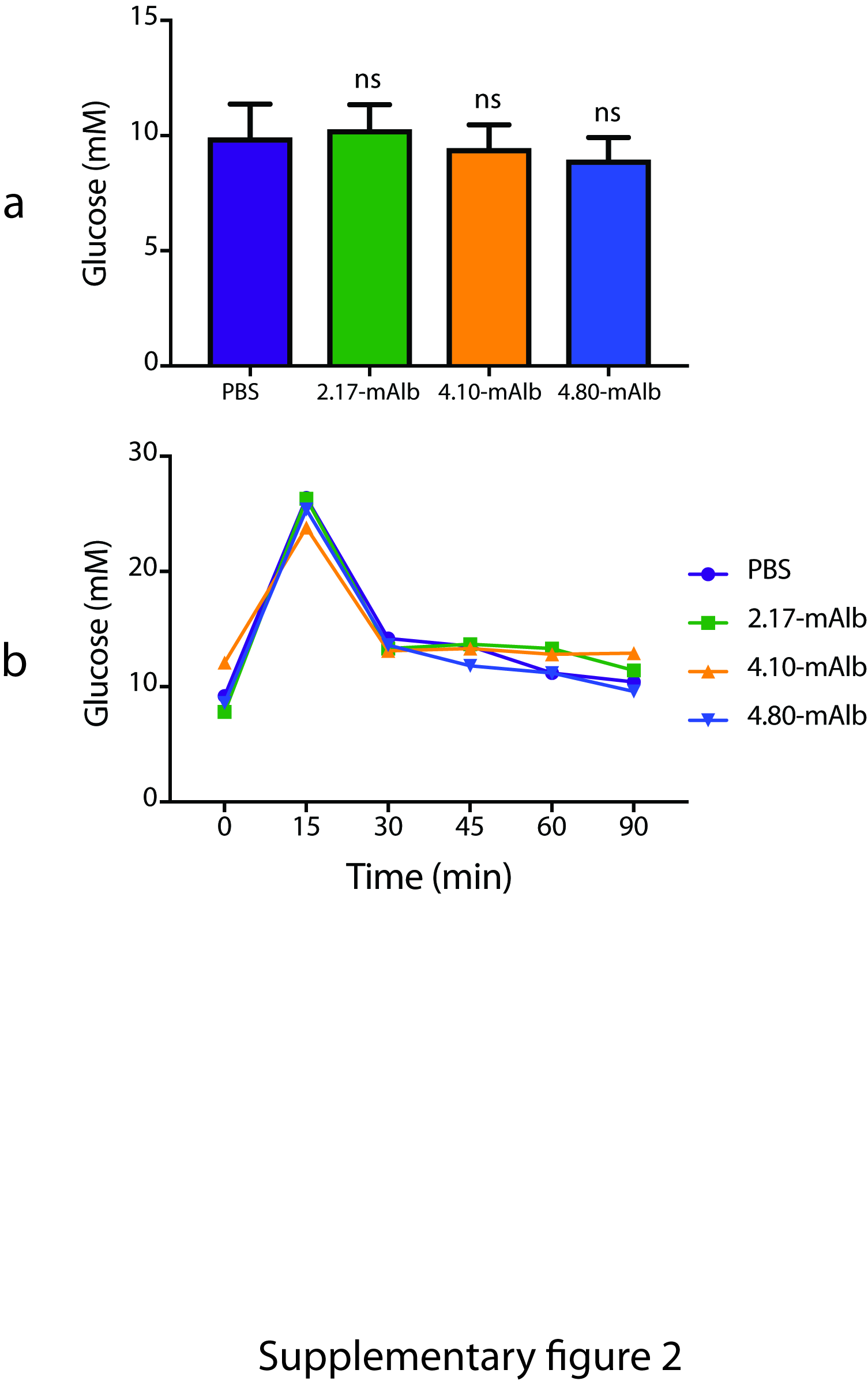

Supplement: Supplementary file 2 — Supplementary Fig. 2. Effect of LR specific VHH’s on basal glucose levels and glucose tolerance C57BL/6 mice were i.p. injected daily with PBS (n = 6) or 200 μg of the mAlb fusions of the LR specific VHH’s 2.17, 4.10 or 4.80 (n = 6) for two weeks. At the end of the experiment, blood was collected for the measurement of basal blood glucose levels (a) and an intraperitoneal glucose tolerance test (IPGTT) (b) was performed after six hours of fasting [file 18_2019_3004_MOESM2_ESM.tif]

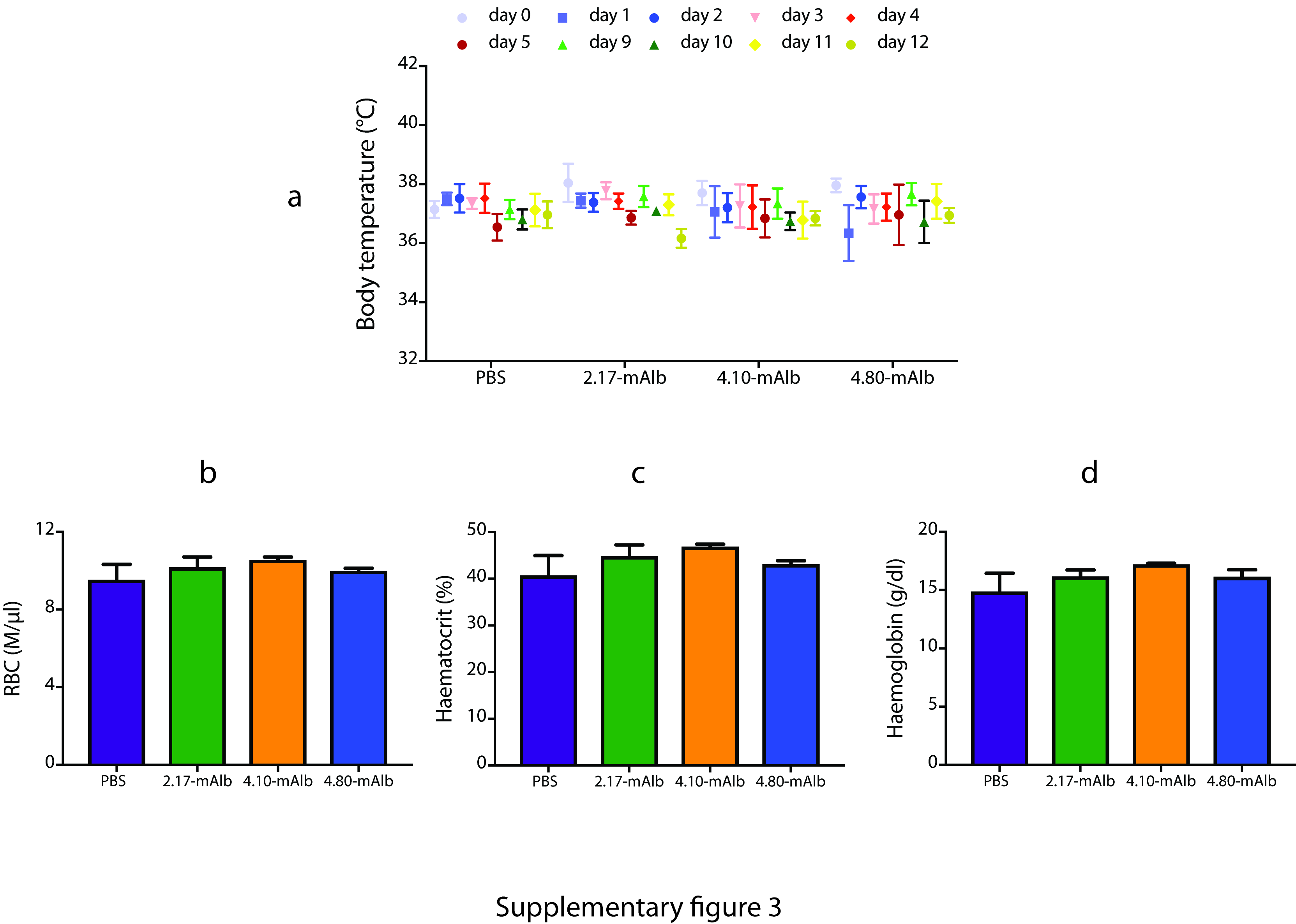

Supplement: Supplementary file 3 — Supplementary Fig. 3. Toxicity assessment of the LR specific VHH’s No toxic side effects were observed based on body temperature (a) during the duration of the experiment or on haematological parameters such as red blood cells numbers (b), haematocrit (c) and haemoglobin concentration (d) at the end of the two-week treatment [file 18_2019_3004_MOESM3_ESM.tif]

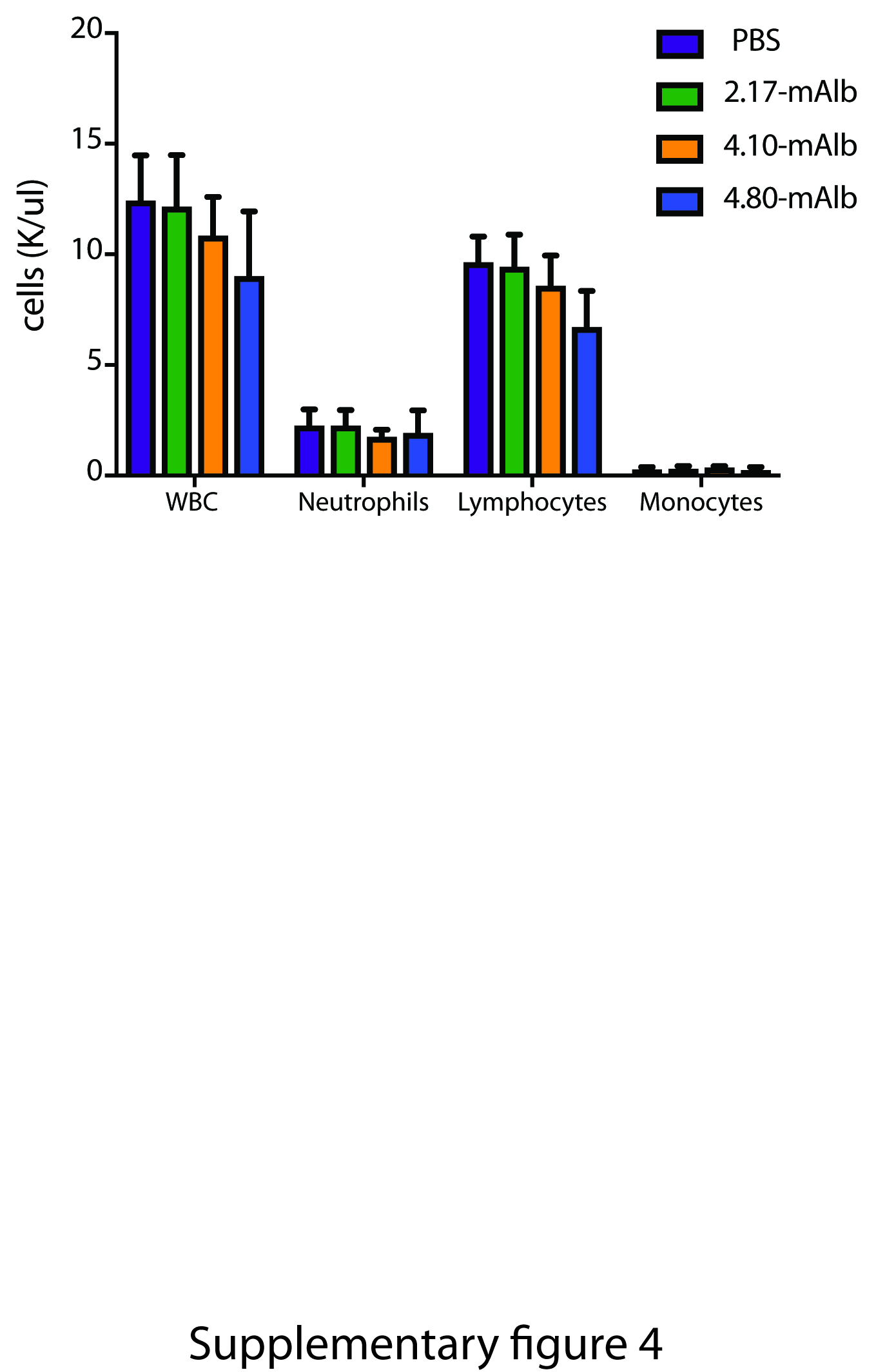

Supplement: Supplementary file 4 — Supplementary Fig. 4. Analysis of white blood cells (WBC) and WBC subtypes Two week treatment with VHH 4.80-mAlb (n = 3) causes partial lymphopenia but not neutropenia. Values represent mean ± SEM; *P < .05, **P < .01, ***P < .005 [file 18_2019_3004_MOESM4_ESM.tif]
